# The Impact of Hydromorphological Alterations on Mayfly Assemblages of a Mid-Sized Lowland River in South-Eastern Europe

**DOI:** 10.3390/insects13050436

**Published:** 2022-05-06

**Authors:** Marina Vilenica, Iva Vidaković Maoduš, Zlatko Mihaljević

**Affiliations:** 1 Faculty of Teacher Education, University of Zagreb, 44250 Petrinja, Croatia; 2 Elektroprojekt d.d., Civil and Architectural Engineering Department, Water Resources, Nature and Environmental Protection Section, Alexandera von Humboldta 4, 10000 Zagreb, Croatia; iva.vidakovic@gmail.com; 3 Division of Zoology, Department of Biology, Faculty of Science, University of Zagreb, Rooseveltov trg 6, 10000 Zagreb, Croatia; zlatko.mihaljevic@biol.pmf.hr

**Keywords:** Ephemeroptera, environmental stress, abiotic parameters, lotic habitats, functional traits

## Abstract

**Simple Summary:**

The majority of European lowland rivers are under the impact of multiple stressors (water quality, hydromorphological alterations, land-use), however, the consequences of these influences on mayflies have not been sufficiently studied. Therefore, we studied mayfly assemblages and their relationship to environmental factors along a mid-sized lowland river in Croatia. No significant differences in mayfly species richness and their functional traits were observed among the three habitat groups with different levels of hydromorphological alterations (near-natural, moderately altered, and severely altered habitats). This could be related to the river’s connection to the numerous tributaries, and the variety of available microhabitats along the studied system, despite the existing hydromorphological pressures. A stronger relationship was found between mayflies and the water physico-chemical characteristics (water temperature, water velocity, oxygen content, and nutrient (e.g., nitrogen, phosphorous) concentrations) and agricultural and urban land-use. These results can contribute to the planning of management and conservation activities for lowland rivers and their biota according to the requirements of the European Water Framework Directive.

**Abstract:**

Historically, rivers have been anthropogenically modified for different purposes worldwide (e.g., flood control, drinking water abstraction, and land drainage). Although the majority of European lowland rivers are under the impact of multiple stressors (water quality, hydromorphological alterations, land-use), the consequences of these influences on aquatic macroinvertebrates, including mayflies, have not been sufficiently studied. Therefore, with the aim of providing additional data on the response of mayflies to anthropogenic disturbances in riverine habitats, we studied mayfly assemblages and their relationship to environmental factors along a mid-sized lowland river in Croatia. No significant differences in mayfly species richness and their functional traits were observed among the three habitat groups with different levels of hydromorphological alterations (near-natural, moderately altered, and severely altered habitats). This could be related to the river’s connection to the numerous tributaries, and the variety of available microhabitats along the studied system, despite the existing hydromorphological pressures. A stronger relationship was found between mayflies and the physico-chemical water characteristics and land-use. Water temperature, water velocity, oxygen content, and nutrient (e.g., nitrogen, phosphorous) concentrations related to agricultural and urban land-use were found to be the most important factors shaping mayfly assemblages in the studied lotic lowland system. These results can contribute to the planning of management and conservation activities for lowland rivers and their biota according to the requirements of the European Water Framework Directive.

## 1. Introduction

Rivers have a long history of being anthropogenically modified for a variety of purposes (e.g., flood control, drinking water abstraction, and land drainage) using a variety of engineering methods, all of which have affected the physical, biological, and downstream river conditions [1]. Recognition that these physical alterations are closely related to aquatic communities has led to the development of several methods for assessing the level of hydromorphological habitat alteration [2,3,4], and confirmation that hydromorphological condition influences benthic macroinvertebrate community structure [5,6,7]. This is particularly important considering the majority of European lowland rivers are under the impact of multiple stressors (water quality, hydromorphological alterations, land-use) [7,8] and that channelization negatively affects the benthic macroinvertebrate diversity of a lowland river, even under low pollution conditions [9].

Mayflies are a small order of amphibious insects with a cosmopolitan distribution [10]. They spend most of their lives as nymphs in almost all types of freshwater habitats, where they are highly dependent on physico-chemical water parameters, such as temperature, water velocity, oxygen content, available nutrients, and substrate type [11,12,13,14,15,16]. Many studies have shown that mayfly assemblages are most diverse in rhithral (upper reaches) sections of fast-flowing streams and rivers, and in pristine large potamal rivers [11,17]. A smaller number of species is adapted to high mountains streams, crenal (spring) sections of streams, and metapotamal river sections (lower reaches) [11]. Mayflies constitute a very large proportion of biomass in freshwater ecosystems [11,18] and are an important food source for a variety of aquatic and terrestrial predators [10]. Depending on the available food resources, different ratios of grazers/scrapers, collectors and filter feeders [10,11] are found in particular habitats. Grazers/scrapers feed mainly on periphyton and fine particulate organic matter (FPOM), while detritivores (collectors, active and passive filter feeders) consume decomposing particulate organic matter (CPOM and FPOM) [11,19,20,21,22,23]. Due to their widespread occurrence, importance in aquatic food webs, and sensitivity to alterations of their habitat e.g., [12,15,16], they are used as indicators of freshwater health in bio-monitoring programs worldwide [10,24].

While it has already been established that hydromorphological degradation leads to several aspects of habitat alteration [25], the extent of these effects on mayflies is not well understood, particularly due to their high drift character [10,26] and the fact that some degradation–such as riprap on banks and channel beds for erosion control–can have both local and downstream geomorphic implications [27]. Many studies have already demonstrated the negative impacts of anthropogenic activities on lotic mayfly assemblages [12,15,16,28,29]. Several mayfly species are already extinct (locally or throughout their range), and many are considered endangered and are therefore listed in national Red Lists [30,31,32]. Ecological assessments of habitats and their biota are essential worldwide to effectively implement conservation and management activities [33,34]. Therefore, with the aim to provide additional data on mayfly response to anthropogenic disturbance in riverine habitats, we studied mayfly assemblages and their relationship to environmental factors along a mid-sized lowland river in south-eastern Europe. The main objectives of this study were: (a) to compare mayfly assemblages at three habitat groups based on the degree of their hydromorphological alteration, (b) to analyze differences in mayfly functional traits among these habitats, (c) to identify the main environmental drivers shaping mayfly assemblages, (d) to analyze the influence of selected environmental variables on mayflies, and (e) to identify the microhabitat preferential choice of recorded species in a studied Pannonian lowland river.

## 2. Materials and Methods

### 2.1. Study Area

The Bednja River is located in the northern part of Croatia and belongs to the Pannonian lowland ecoregion (ER11). It is the largest tributary of the Drava River in Croatia, with a catchment area of about 600 km^2^. According to Water Framework Directive, Bednja River is categorized as a mid-sized river, with its upper reaches belonging to the small stream type. The river has a relatively small altitude drop from the source to the mouth, which is about 175 m along the entire river length of 105 km. The basin has a temperate humid climate, corresponding to type Cfb by Köppen climate classification, where the average temperature in the warmest month does not exceed 22 °C and the average temperature in the coldest month does not fall below −3 °C [35]. The average monthly air temperature in the study area in the year of the study (2015) was 11.6 °C (±7.6 °C) and the average total annual precipitation was 897 mm (±114.6 mm) (source: personal calculation from raw data obtained upon request from the Croatian Meteorological and Hydrological Service). The discharge regime of the river is classified as peripannonian pluvial-nival [36].

**Figure 1:**
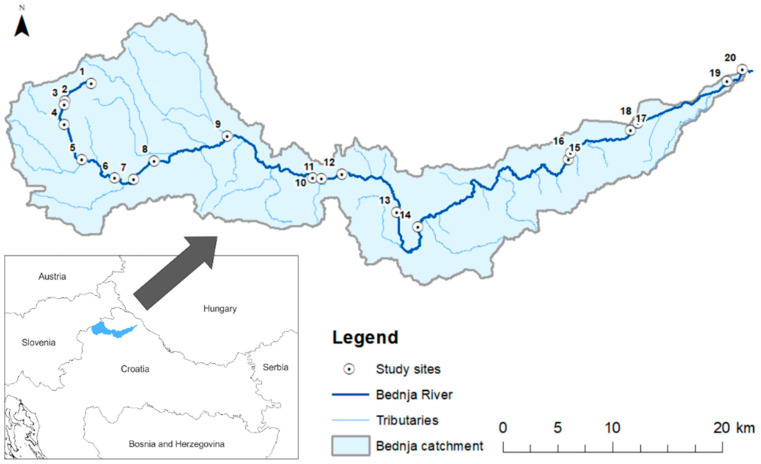
Spatial distribution of the study sites and associated sub-catchments along the Bednja River and position of the catchment in northern Croatia (Pannonian lowland ecoregion, ER11).

Extensive agriculture in the form of pasture, and intensive agriculture in the form of complex cropping, are the most common types of agriculture practiced in the catchment. At the time of this study, the villages and towns along the river did not have wastewater treatment. The 20 suitable study sites (Figure 1) were selected for their representativeness of different conditions and stressors, including areas upstream and downstream of wastewater outlets, and different level of morphological alteration.

### 2.2. Environmental Variables

Water quality parameters were measured at each study site on three occasions: spring (20–24 March), summer (28 June–7 July), and autumn (4 October) 2015. The following parameters were measured *in situ*: water temperature and oxygen saturation (using the oximeter WTW Oxi 330/SET), pH value (using the pH meter WTW pH 330), and conductivity (using the conductometer WTW LF 330). The other parameters and nutrients were analyzed in the laboratory according to standard methods [37]: biological oxygen demand, chemical oxygen demand, concentrations of total nitrogen, nitrites, nitrates, organic nitrogen, Kjeldahl nitrogen, ammonium, total phosphorous, and orthophosphates.

For each study site, geographical attributes (distance from source) and land-use percentage (using CORINE Land Cover as a layer [38]) were calculated using GIS tools Arc Map version 10 [39]. Land use variables were defined from the share of land use categories at the catchment scale, and the categories were combined into four land use variables: near-natural, urban, intensive agricultural, and extensive agricultural land-use.

The extent of hydromorphological alteration was assessed based on the European Standard EN 15843:2010 [40]. The assessment was performed for a reach length of 500 m, which includes the sampling site and extends upstream. Although the EN 15843:2010 methodology assesses hydromorphological alteration that includes morphological features, flow regime, and longitudinal continuity, for this study we used only the score for morphological modification as a variable.

**Figure 2:**
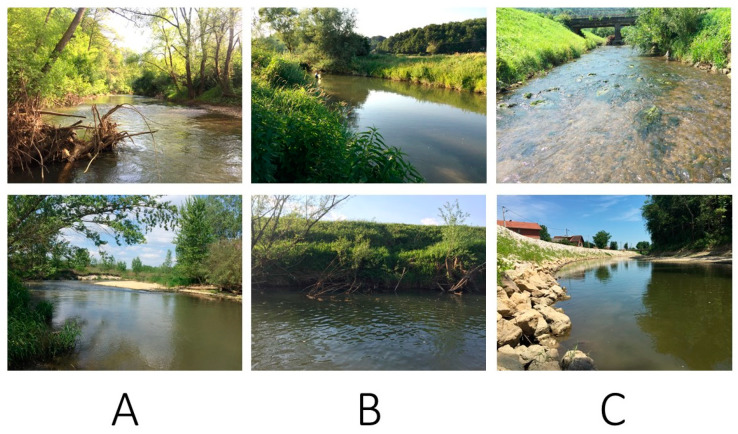
Examples of the three morphological habitat types in a Pannonian lowland river: (**A**)—study sites 16 (**top**) and 20 (**bottom**), with natural riparian vegetation and little or no physical alteration to the banks, no artificial substrate, and predominantly semi-natural surrounding land-use. (**B**)—study sites 10 (**top**) and 15 (**bottom**), with resectioned and realigned channel, steep banks with predominantly grass vegetation and individual trees, and agricultural land-use in surrounding areas. (**C**)—study sites 7 (**top**) and 19 (**bottom**), with modified channel with riprap reinforcement, and predominantly surrounding urban land-use.

The final score for morphological modification represents the average score of assessed features: channel planform and section, extent of artificial material and alteration of natural substrate character, bank structure and modifications, vegetation type/structure on banks and adjacent land, adjacent land-use, degree of lateral connectivity of river and floodplain, and degree of lateral movement of river channel. Based on these scores, three main habitat groups were identified: group A (score < 2.5; near-natural to slightly altered sites), group B (score between 2.5 and 3.5; slightly to moderately altered sites), and group C (score ˃ 3.5; extensively to highly altered sites) (Table 1, Figure 2).

### 2.3. Mayfly Sampling

Mayflies were sampled in summer 2015 (30 June, 1, 3, 4, 5, and 7 July) with a hand net (0.5 mm mesh size)–together with other benthic macroinvertebrates–using the “multihabitat method”, as presented in the AQEM manual [41]. At each site, a total of 20 longitudinally distributed subsamples (dominant microhabitats, covering > 5% of each site) were collected, covering approximately 1.25 m^2^ of the stream bottom area. During sampling, individual subsamples were grouped by microhabitat in separate containers and then preserved in 96% ethanol. Substrates at the sampling sites were categorized according to AQEM consortium [41] (Supplementary Table S1). Samples were consequently sorted in the laboratory. Mayflies were identified to the lowest possible taxonomic level (very juvenile and/or damaged individuals were identified only to genus or family level) using relevant identification keys [42,43,44]. The voucher specimens are deposited in the first author’s collection, at the Department of Biology, Faculty of Science, University of Zagreb, Croatia.

### 2.4. Data Analyses

The Kruskal-Wallis H test and Multiple comparisons *post hoc* test were applied to determine differences in water velocity and water depth among different microhabitats. The same testing was used to determine species associations with substrate types in the microhabitats studied, treating subsamples as replicates. This analysis was performed in Statistica, version 10.0 [45].

Spearman’s rank correlation coefficient (R) was used to analyze the relationship between mayfly assemblages (abundance (number of individuals per m^2^), taxa richness) and each of the recorded species with physico-chemical water properties, land-use, and the score for morphological modification. The same test was used to determine correlations between mayfly species and water velocity and depth in studied microhabitats. Subsamples were treated as replicates. This analysis was performed in Statistica, version 10.0 [45].

Non-metric multidimensional scaling analysis (NMDS) was applied to reveal similarities in the composition of mayfly assemblages among the three habitat type groups (with study sites as replicates) using the Bray–Curtis similarity index. The results of the hierarchical cluster analysis are superimposed on NMDS ordination. These analyses were performed using Primer 6 software package [46].

The composition of mayfly assemblages in terms of the functional feeding guilds and longitudinal distribution associations of species at three habitat groups was categorized using data from Buffagni et al. [22]. The functional feeding guild and longitudinal zonal association of each species are presented as a proportion within the assemblage. Most mayfly species do not feed exclusively on a single food resource and do not occur exclusively in one biocenotic region; therefore, assignment of species to a particular category is based on the 10-point assignment scale (see [23]). Therefore, we calculated the functional feeding-guild composition and the longitudinal zonation associations of mayfly assemblages in each of the habitat groups (treating the study sites as replicates), using the given points and percentage of each species within the assemblage.

To ordinate mayfly occurrence in relation to environmental variables, Canonical Correspondence Analysis (CCA) was used. The analysis was performed using subsample data for 26 taxa (taxa data were log-transformed, rare species were downweighed) and 11 environmental variables (significant variables were selected using Interactive-forward-selection (distance from source, water temperature, oxygen saturation, water velocity, water depth, concentration of orthophosphates, ammonium, nitrites, total nitrogen, intensive land use, urban land use)). The Monte Carlo permutation test with 499 permutations was used to test the statistical significance of the relationship between all taxa and selected variables. The CCA was performed using CANOCO 5.00 [47].

## 3. Results

### 3.1. Environmental Variables

**Figure 3:**
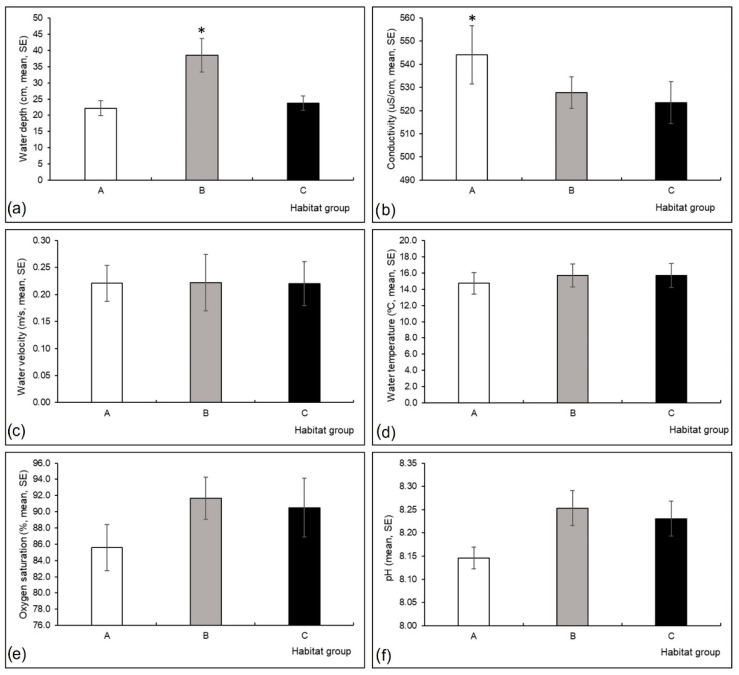
Mean (and standard error, SE) values of environmental variables measured at three habitat groups in a Pannonian lowland river: (**a**) water depth (cm), (**b**) conductivity (µS/cm), (**c**) water velocity (m/s), (**d**) water temperature (°C), (**e**) oxygen saturation (%), (**f**) pH. Asterisk is for significant results. Habitat groups (A, B, C) are defined in Table 1.

**Figure 4:**
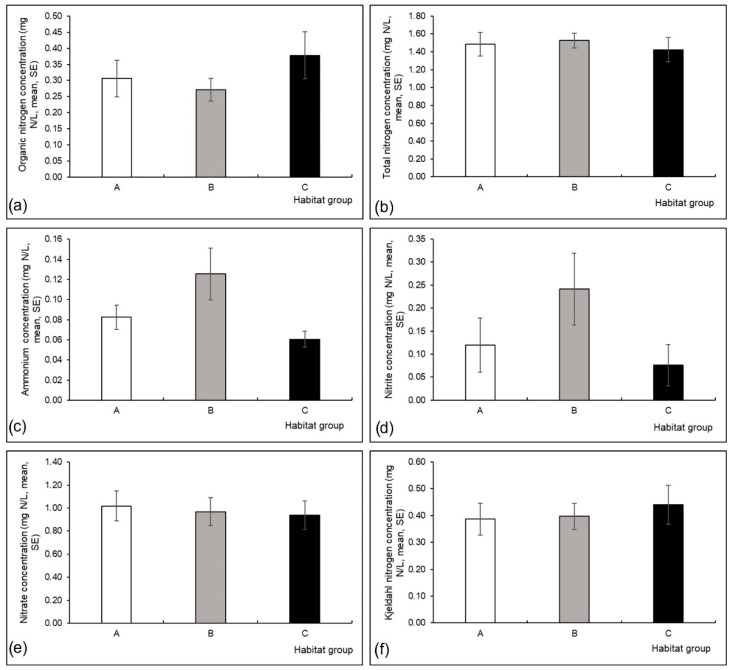
Mean (and standard error, SE) values of environmental variables measured at three habitat groups in a Pannonian lowland river: (**a**) organic nitrogen concentration (mg N/L), (**b**) total nitrogen concentration (mg N/L), (**c**) ammonium concentration (mg N/L), (**d**) nitrite concentration (mg N/L), (**e**) nitrate concentration (mg N/L), (**f**) Kjedahl nitrogen concentration (mg N/L). Habitat groups (A, B, C) are defined in Table 1.

**Figure 5:**
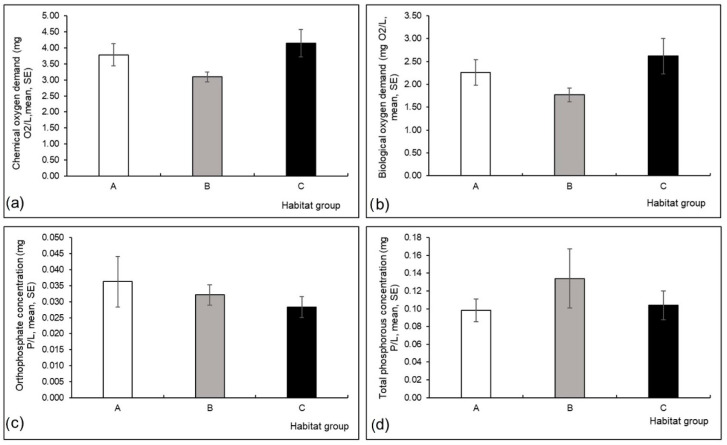
Mean (and standard error, SE) values of environmental variables measured at three habitat groups in a Pannonian lowland river: (**a**) chemical oxygen demand (mg O_2_/L), (**b**) biological oxygen demand (mg O_2_/L), (**c**) orthophosphate concentration (mg P/L), (**d**) total phosphorous concentration (mg P/L). Habitat groups (A, B, C) are defined in Table 1.

Conductivity (Kruskal-Wallis H test, H (2, N = 60) = 7.40, *p* = 0.03) and water depth (H (2, N = 60) = 6.68, *p* = 0.04) differed significantly among the three habitat groups; conductivity was higher in group A, and water depth was higher in group B compared to the other habitat groups (Figure 3a,b). Differences in other measured environmental variables were not significant among the three habitat groups (Figure 3, Figure 4 and Figure 5).

### 3.2. Water Velocity and Water Depth in Studied Microhabitats

**Figure 6:**
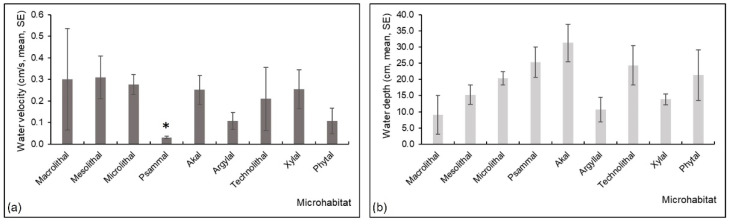
Mean (and standard error, SE) values of (**a**) water velocity and (**b**) water depth in various microhabitats in a Pannonian lowland river. Asterisk is for significant results.

The Kruskal-Wallis H test showed there were differences in water velocity among different substrate types in the studied lotic system (H (8, N = 71) = 28.03, *p* = 0.0005). According to the Multiple Comparisons *post hoc* test, psammal substrate had significantly lower water velocity compared to mesolithal (*p* = 0.02), microlithal (*p* = 0.02), akal (*p* = 0.006), and xylal (*p* = 0.03) (Figure 6a). No significant differences were found in water depth among substrates, but higher depth was recorded on psammal and akal compared to the other substrates (Figure 6b).

### 3.3. Mayfly Assemblages

A total of 22 species was identified (Table 2). *Baetis fuscatus*, *Baetis buceratus*, *Baetis rhodani*, *Baetis vernus*, *Procloeon bifidum*, *Heptagenia flava*, *Habrophlebia lauta*, *Serratella ignita*, *Caenis luctuosa,* and *Caenis* cf. *pseudorivulorum* were present at all three habitat groups, with *Baetis fuscatus* being the most abundant (Table 2). *Cloeon dipterum* was the rarest species, occurring in low abundance only in habitat group C (Table 2).

**Figure 7:**
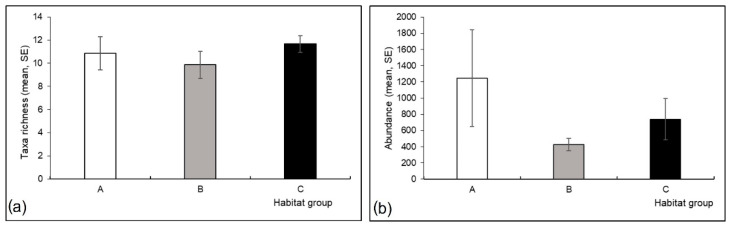
Mayfly assemblages at three studied habitat groups in a Pannonian lowland river: (**a**) taxa richness and (**b**) abundance (number of individuals per m^2^) (shown as mean with standard error, SE). Habitat groups (A, B, C) are defined in Table 1.

No significant differences were found among the three habitat groups in terms of taxa richness (Kruskal-Wallis H test; H (2, N = 19) = 0.36, *p* = 0.84) and abundance (H (2, N = 19) = 0.84, *p* = 0.66). Nevertheless, taxa richness and abundance were lower in group B compared to the other two groups (Figure 7).

**Figure 8:**
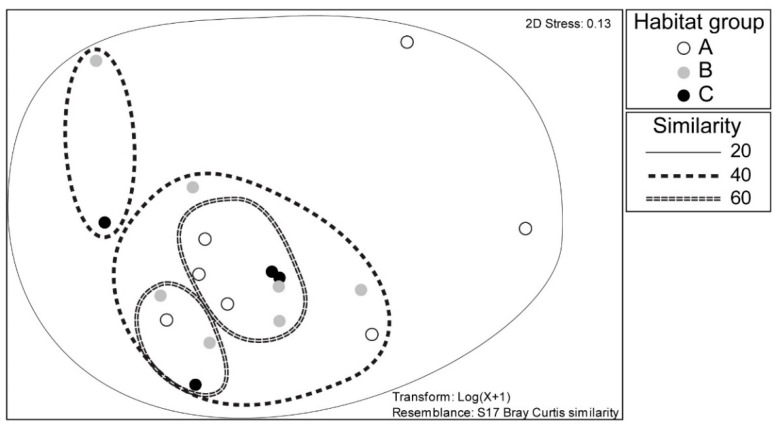
NMDS analysis of the study sites belonging to three habitat type groups in a Pannonian lowland river, based on the composition of mayfly fauna. Habitat groups (A, B, C) are defined in Table 1.

In NMDS analysis, mayfly assemblages mostly did not group based on the habitat group they belong to (Figure 8).

**Figure 9:**
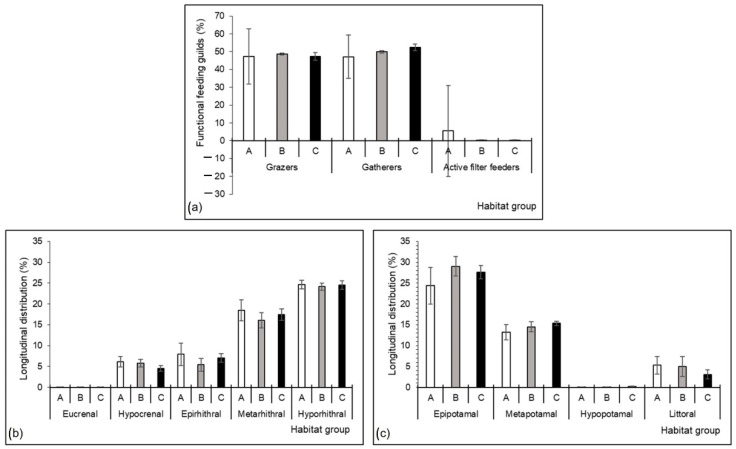
Mayfly assemblage structure at three habitat groups in a Pannonian lowland river (shown as mean with standard error, SE): (**a**) functional feeding guilds, and (**b**,**c**) longitudinal distribution associations. Habitat groups (A, B, C) are defined in Table 1.

The structure of mayfly assemblages was similar in all three habitat groups (Figure 9). Compared to the other habitat groups, there was a slightly higher proportion of grazers in group B, gatherers in group C, and active filter feeders in group A (Figure 9a). At all three sites, a domination of species characteristic for hyporhithral and epipotamal river sections were recorded (Figure 9b,c). Group A had a slightly higher proportion of species associated with rhithral river sections (Figure 9b), while groups B and C had a slightly higher proportion of species associated with potamal river sections (Figure 9c).

### 3.4. Mayflies and Environmental Variables

Positive correlations of mayfly taxa richness were found with water temperature (R = 0.38, *p* = 0.001), oxygen saturation (R = 0.33, *p* = 0.004), pH (R = 0.24, *p* = 0.04), biological oxygen demand (R = 0.25, *p* = 0.03), chemical oxygen demand (R = 0.25, *p* = 0.03), nitrate concentration (R = 0.29, *p* = 0.01), and total nitrogen (R = 0.24, *p* = 0.04). Negative correlations were found between mayfly taxa richness and near natural land-use (R = −0.34, *p* = 0.004), while positive correlations were found with morphological modification score (R = 0.36, *p* = 0.002), distance from source (R = 0.29, *p* = 0.01), and extensive agriculture (R = 0.29, *p* = 0.01).

Positive correlations of mayfly abundance were recorded with oxygen saturation (R = 0.39, *p* = 0.001), water temperature (R = 0.36, *p* = 0.002), water velocity (R = 0.32, *p* = 0.01), pH (R = 0.33, *p* = 0.01), nitrate concentration (R = 0.27, *p* = 0.02), and total nitrogen (R = 0.26, *p* = 0.03). Mayfly abundance negatively correlated with near natural land-use (R = −0.26, *p* = 0.03), while positive correlations were found for extensive agriculture (R = 0.31, *p* = 0.01) and distance from source (R = 0.31, *p* = 0.01).

Abundance of majority of recorded mayfly species showed significant correlations with measured water quality parameters (Table 3 and Table 4) and natural and anthropogenic variables (Table 5).

**Figure 10:**
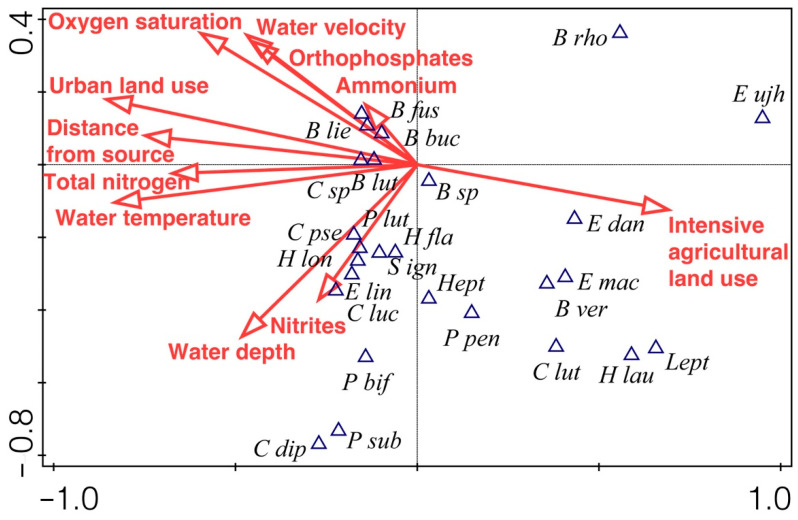
F1 × F2 plane of Canonical correspondence analysis (CCA) based on 26 mayfly taxa and 11 environmental variables in a Pannonian lowland river. For taxa code abbreviations (triangle symbols) see Table 2.

The results of the ordination of species and environmental data of the CCA are presented on the F1 × F2 ordination plot (Figure 10). The eigenvalues for the first two CCA axes were 0.49 and 0.31, explaining 27.4% of the species–environment relations. The Monte Carlo permutation test showed that the species–environment ordination was significant (first axis: F-ratio = 10.64, *p* = 0.002; overall: trace = 1.44, F = 3.52, *p* = 0.002), indicating that mayfly assemblages were significantly related to the tested set of environmental variables. Axis 1 was related to urban land use (R = −0.80) and water temperature (R = −0.78), and axis 2 to water depth (R = −0.80) and nitrite concentration (R = −0.32), indicating that these were the most important parameters in explaining patterns of mayfly assemblages (Figure 10).

### 3.5. Mayfly Species and Microhabitats

*Baetis fuscatus* was significantly more abundant in microlithal compared to psammal (Kruskal-Wallis H test and Multiple comparisons *post hoc* test; H (8, N = 62) = 21.25, *p* = 0.007), while *Heptagenia flava* was significantly more abundant in xylal compared to akal (H (8, N = 35) = 23.86, *p* = 0.002).

*Baetis fuscatus* was more abundant in microhabitats with higher water velocity (R = 0.62, *p* = 0.000) and water depth (R = 0.23, *p* = 0.05), *Baetis rhodani* was more abundant in microhabitats with higher water velocity (R = 0.31, *p* = 0.008) and lower water depth (R = −0.28, *p* = 0.02), and *Procloeon bifidum* was more abundant in microhabitats with lower water velocity (R = −31, *p* = 0.007) and higher water depth (R = 0.32, *p* = 0.006).

Microhabitats with lower water depth were associated with *Baetis vernus* (R = −0.35, *p* = 0.003) and *Electrogena ujhelyii* (R = −0.40, *p* = 0.0005), and microhabitats with higher water depth were associated with *Caenis luctuosa* (R = 0.35, *p* = 0.002), *Caenis* cf. *pseudorivulorum* (R = 0.33, *p* = 0.004), *Ephemera lineata* (R = 0.36, *p* = 0.002), and *Potamanthus luteus* (R = 0.44, *p* = 0.0001).

*Centroptilum luteolum* (R = −0.23, *p* = 0.049) and *Cloeon dipterum* were recorded at microhabitats with lower water velocity (R = −0.32, *p* = 0.008), while *Serratella ignita* was associated with microhabitats with higher water velocity (R = 0.38, *p* = 0.001).

## 4. Discussion

Despite the present hydromorphological alterations, with approximately a quarter (26%) of the Croatian mayfly fauna [48], mayfly assemblages in the studied lotic system can be considered species-rich when compared to some other anthropogenically impacted lowland lotic habitats [16,49]. However, this could be a result of the sampling effort, which included a large number of study sites along the Bednja River system. The most abundant and widespread species was *Baetis fuscatus*, a lotic species commonly found in the main river channel of lowland running waters [16,22]. *Cloeon dipterum*–a eurytopic species with lentic preference, characterized by high ecological tolerance to habitat disturbance [22,50]–was the rarest in our study, occurring only in habitats with the highest degree of hydromorphological degradation, where it was associated with microhabitats with lower water velocity. Interestingly, no representatives of the genera *Rhithrogena* and *Epeorus* were found in our study. This could be due to the late sampling period, but also to the range of water velocity and substrate composition which are not optimal for representatives of these taxa as they are mostly litho-rheobiontic, i.e., they usually prefer riffle zones of fast-flowing streams with stony bottoms [11,21,22]. In addition, most *Rhithrogena* species have a rather low tolerance to organic pollution [11], which could have also influenced their absence in the studied river system.

No relevant differences were found among the three hydromorphological habitat groups in abiotic parameters, mayfly assemblage composition, or functional traits, although mayfly abundance was slightly higher in the non-altered habitat group. Previous studies have shown that anthropogenic disturbances such as river channelization, impoundment, eutrophication, pollution, and microhabitat homogeneity have negative effects on assemblages of aquatic insects, including mayflies [12,15,16,51,52]. No significant differences in taxa richness among hydromorphological habitat groups could be partially explained by high drifting potential of mayfly nymphs [26]. For mayflies, drift may represent a means by which nymphs move to more optimal habitats, e.g., by colonization of new downstream sites, especially following floods or pollution events [53]. The lotic system studied includes a mosaic of near-natural sites, and those with moderate or severe hydromorphological alterations. Therefore, some of the mayfly species collected in low numbers in highly altered habitat group (e.g., *Baetis liebenauae, Ecdyonurus macani, Paraleptophlebia submarginata*) may have been collected during their downstream drift to more suitable habitats, or might have drifted into the main river channel from some of the numerous tributaries connected to the Bednja River. However, this should be further investigated in future studies with more frequent (i.e., monthly) sampling efforts. The Bednja River system still has a relatively diverse microhabitat composition at most study sites, which may have provided adequate conditions for the mayflies recorded despite the challenges of habitat hydromorphological alterations they face, similar to the study by Vilenica et al. [12].

However, some differences among habitat groups were observed at the species level: several species with moderate (e.g., *Baetis fuscatus*, *Serratella ignita*, *Electrogena ujhelyii*) to strong rhithral affinity (*Baetis vernus*, *Habrophlebia lauta*) [22,54] were predominantly associated with near-natural sites (with little or no hydromorphological degradation), while some eurytopic (e.g., *Baetis rhodani*) and species characteristic for potamal river regions and lentic habitats (e.g., *Cloeon dipterum*, *Procloeon bifidum*, *Centroptilum luteolum*, *Caenis luctuosa*) were more abundant at sites with moderate or severe hydromorphological alteration [22,55,56].

Our results suggest that mayflies are more strongly related to physico-chemical water parameters along the longitudinal gradient and microhabitat heterogeneity than with hydromorphological alterations in the studied lotic system. The study by Vidaković Maoduš et al. [57] showed that there is an increasing longitudinal gradient in some of the measured water parameters (water temperature, oxygen saturation, water velocity, pH, nutrients) along the Bednja River system. Therefore, we observed a link between higher mayfly species richness and abundance in the studied lotic system and higher water temperature and oxygen saturation, higher water velocity and lower depth, and higher nutrient concentration (e.g., various nitrogen forms). For this reason, mayfly assemblages were also significantly related to distance from the river source and extensive agricultural and urban land-use. Mayflies can be found at both lotic and lentic habitats, although a higher number of species are restricted to running waters [11,15,50]. In lotic habitats, the mayfly species richness is lowest in sources of streams and rivers–habitats characterized by low water temperatures. Their assemblages tend to be more diverse with the longitudinal increase in water temperature [12,13,17,58]. In addition, most mayfly species require well-oxygenated water [11,12,13], which is often associated with higher water velocity in lotic habitats. Associations with abiotic parameters were also observed at the species level, e.g., several species that prefer moderate and warm water (>10 °C), such as *Baetis buceratus* and *Procloeon bifidum*, were associated with habitats with warmer water [21,22]. Some eurytherm species (such as *Baetis fuscatus*, *Baetis liebenauae*, *Baetis lutheri*, *Cloeon dipterum*, *Heptagenia longicauda*, *Potamanthus luteus*, *Serratella ignita*, *Caenis* cf. *pseudorivulorum*, and *Caenis luctuosa*) correlated positively with water temperature, while some of them (e.g., *Baetis vernus*, *Habrophlebia lauta*) [21,22] were associated with habitats with lower water temperature. The rheo to limnophil *Baetis fuscatus*, *Baetis buceratus*, *Baetis liebenauae*, and *Serratella ignita* were associated with sites with higher water velocity, while the limnophil *Centroptilum luteolum*, *Cloeon dipterum*, *Electrogena ujhelyii*, the limno to rheophile *Habrophlebia lauta*, and the rheo to limnophil *Procloeon bifidum* were associated with sites with lower water velocity [21,22,59].

Higher nutrient concentrations derived from agricultural fields and urban areas in the Bednja River catchment may have enhanced primary production (e.g., higher macrophyte and algae development) in the studied system [60], providing more food for grazers [11,14], which, along with collectors, were the dominant feeding guild at all three habitat groups. Nevertheless, increasing nutrient inputs to the aquatic habitats may have long-term negative consequences, leading to severe eutrophication that could negatively influence inhabiting biota [61]. *Baetis buceratus*, *Baetis fuscatus*, *Heptagenia longicauda*, *Potamanthus luteus*, *Serratella ignita*, *Caenis* cf. *pseudorivulorum*, and *Caenis luctuosa* showed positive correlations with nutrient concentrations in water, indicating their tolerance to water pollution. On the other hand, the negative associations of *Baetis rhodani*, *Baetis vernus*, *Procloeon pennulatum*, *Electrogena ujhelyii*, and *Habrophlebia lauta* with nutrients in their habitats could indicate their sensitivity to water pollution and eutrophication.

In the studied system, most species that showed a significant association with specific microhabitat components (substrate type, water velocity and/or depth) are known to be generalists, i.e., they are found on different substrates [21,22]. This was confirmed in our study, as most of these species were not associated with a specific substrate type, but with the water depth and/or velocity at the available microhabitats. However, some of them were associated with specific substrates, such as *Heptagenia flava*, which was most abundant at microhabitats with xylal, and *Baetis fuscatus*, which was most abundant in microhabitats with microlithal, higher water velocity and water depth. We also recorded several microhabitat specialists, such as *Ephemera lineata*–a specialist for microhabitats with psammal [21,22]–which was most abundant at microhabitats with higher water depth, associated with fine substrate in the studied system. *Centroptilum luteolum*, a specialist for microhabitats with macrophytes [21,22], was most abundant in microhabitats with lower water velocity, but mostly associated with different inorganic substrates. Microhabitat heterogeneity, in addition to physical and chemical water properties, is another key factor that shapes mayfly assemblages [14,62,63], as it allows their nymphs to seek refuge from predators and find sufficient food resources [14,64,65]. Organic substrates (especially macrophytes) and coarse inorganic sediment are important habitat segments for nymphs of various mayfly species [11,14,17], as they trap organic matter and provide a habitat for periphyton [65,66,67], which is an important food resource for mayfly nymphs.

## 5. Conclusions

Hydromorphological habitat modifications are known to have negative consequences on mayflies in lotic habitats, however, in our study, more relevant differences in species composition resulted from the interplay of water abiotic factors and catchment land-use. Moreover, despite the hydromorphological alterations, the high number of tributaries and the microhabitat heterogeneity observed along the studied river may have influenced the relatively high mayfly species richness, not only in near-natural sites, but also in those belonging to moderately and highly modified habitat groups. To successfully assess the environmental quality of riverine habitats, it is essential to obtain detailed knowledge of their assemblages, and the relationship between the biota and environmental variables. Mayflies are among the most commonly used taxa in biomonitoring programs worldwide due to their bioindicative properties. Therefore, our results can contribute to the planning of management and conservation activities for lowland rivers and their biota according to the requirements of the European Water Framework Directive.

## Figures and Tables

**Table 1:** Investigated sites along a Pannonian lowland river, morphological modification scores, corresponding habitat group: Group A (score < 2.5; near-natural to slightly altered sites), group B (score between 2.5 and 3.5; slightly to moderately altered sites), and group C (score ˃ 3.5; highly to very highly altered sites), number of dominant microhabitats present at each site (covering > 5% of the site; see also Supplementary Table S1) and number of recorded mayfly taxa.

Study Site	Morphological Modification Score	Habitat Group	Number of Microhabitats	Number of Mayfly Taxa
1	1.78	A	6	4
2	3.00	B	3	7
3	2.22	A	6	10
4	1.44	A	3	5
5	3.89	C	3	7
6	4.56	C	4	11
7	4.22	C	4	8
8	2.78	B	3	7
9	2.78	B	3	5
10	3.22	B	2	8
11	4.11	C	3	12
12	2.89	B	4	13
13	2.89	B	4	8
14	2.11	A	3	10
15	2.89	B	3	11
16	1.67	A	5	12
17	5.00	C	2	11
18	2.44	A	4	10
19	3.67	C	2	10
20	1.22	A	4	10

**Table 2:** Mayfly taxa and their abundance (number of individuals per m^2^) recorded at three habitat groups in a studied Pannonian lowland river. Taxa codes are those used in the CCA analysis. Habitat groups (A, B, C) are defined in Table 1.

Mayfly Taxa/Habitat Group	Taxa Codes	A	B	C
*Baetis* sp.	*B* sp.	200	100	159
*Baetis fuscatus* (Linnaeus, 1761)	*B fus*	610	105	266
*Baetis buceratus* Eaton, 1870	*B buc*	148	28	114
*Baetis rhodani* (Pictet, 1843)	*B rho*	42	37	71
*Baetis vernus* Curtis, 1834	*B ver*	34	16	19
*Baetis liebenauae* Keffermüller, 1974	*B lie*	4		5
*Baetis lutheri* Müller-Liebenau, 1967	*B lut*		1	9
*Centroptilum luteolum* Müller, 1776	*C lut*	2	4	
*Cloeon dipterum* (Linnaeus, 1761)	*C dip*			1
*Procloeon bifidum* (Bengtsson, 1912)	*P bif*	1	2	5
*Procloeon pennulatum* Bengtsson, 1915	*P penn*	1		1
Heptageniidae	*Hept*	5	4	2
*Ecdyonurus macani* Thomas & Sowa, 1970	*E mac*			3
*Electrogena ujhelyii* (Sowa, 1981)	*E ujh*	22		1
*Heptagenia flava* Rostock, 1878	*H fla*	2	1	4
*Heptagenia longicauda* (Stephens, 1836)	*H lon*	1	1	
Leptophlebiidae	*Lept*	8		1
*Habrophlebia lauta* McLachlan, 1884	*H lau*	29	2	2
*Paraleptophlebia submarginata* (Stephens, 1836)	*P sub*			2
*Ephemera danica* Müller, 1764	*E dan*	2		
*Ephemera lineata* Eaton, 1870	*E lin*	1	1	
*Potamanthus luteus* (Linnaeus, 1767)	*P lut*	1	1	2
*Serratella ignita* (Poda, 1761)	*S ign*	121	71	69
*Caenis* sp.	*C* sp.	2		1
*Caenis luctuosa* (Burmeister, 1839)	*C luc*	9	39	6
*Caenis* cf. *pseudorivulorum* Keffermüller, 1960	*C pse*	3	3	2
Abundance (individuals/m^2^)		1247	415	739
Taxa richness		22	17	22

**Table 3:** Correlations of mayfly species abundance and environmental parameters measured *in situ* in a Pannonian lowland river. Positive correlations are shaded. Only significant results are shown.

Mayfly Taxa/Abiotic Parameter	Water Temperature (°C)	O_2_ %	Conductivity (µS/cm)	pH	Water Depth (cm)	Water Velocity (m/s)
*Baetis fuscatus*	R = 0.57,	R = 0.54,	R = −0.25,	R = 0.58,		R = 0.67,
	*p* = 0.000	*p* = 0.000	*p* = 0.03	*p* = 0.000		*p* = 0.000
*Baetis buceratus*	R = 0.33,					R = 0.25,
	*p* = 0.004					*p* = 0.04
*Baetis rhodani*					R = −0.56,	
					*p* = 0.01	
*Baetis vernus*	R = −0.28,				R = −0.26,	
	*p* = 0.02				*p* = 0.03	
*Baetis liebenauae*	R = 0.31,					R = 0.25,
	*p* = 0.01					*p* = 0.04
*Baetis lutheri*	R = 0.28,	R = 0.38,	R = −0.31,	R = 0.31,		
	*p* = 0.02	*p* = 0.001	*p* = 0.01	*p* = 0.01		
*Centroptilum luteolum*		R = −0.24,		R = −0.25,		R = −0.25,
		*p* = 0.04		*p* = 0.03		*p* = 0.03
*Cloeon dipterum*	R = 0.33,				R = 0.29,	R = −0.34,
	*p* = 0.004				*p* = 0.01	*p* = 0.004
*Procloeon bifidum*	R = 0.25,				R = 0.39,	R = −0.33,
	*p* = 0.04				*p* = 0.001	*p* = 0.01
*Electrogena ujhelyii*	R = −0.53,		R = 0.48,	R = −0.37,	R = −0.63,	R = −0.26,
	*p* = 0.000		*p* = 0.000	*p* = 0.002	*p* = 0.000	*p* = 0.03
*Heptagenia longicauda*	R = 0.32,					
	*p* = 0.01					
*Habrophlebia lauta*	R = −0.27,			R = −0.28,	R = −0.24,	R = −0.29,
	*p* = 0.03			*p* = 0.02	*p* = 0.04	*p* = 0.02
*Ephemera lineata*					R = 0.34,	
					*p* = 0.003	
*Potamanthus luteus*	R = 0.48,	R = 0.33,		R = 0.29,	R = 0.29,	
	*p* = 0.000	*p* = 0.01		*p* = 0.01	*p* = 0.02	
*Serratella ignita*	R = 0.60,	R = 0.51,		R = 0.43,	R = 0.31,	R = 0.28,
	*p* = 0.000	*p* = 0.000		*p* = 0.0001	*p* = 0.01	*p* = 0.02
*Caenis luctuosa*	R = 0.44,	R = 0.25,		R = 0.27,	R = 0.26,	
	*p* = 0.0001	*p* = 0.03		*p* = 0.02	*p* = 0.03	
*Caenis* cf. *pseudorivulorum*	R = 0.25,				R = 0.43,	
	*p* = 0.03				*p* = 0.0002	

**Table 4:** Correlations of mayfly species abundance and environmental parameters analysed in the laboratory. Positive correlations are shaded. Abbreviations: BOD = biological oxygen demand (mg O₂/L), COD = chemical oxygen demand (mg O₂/L), NH₄⁺ = ammonium concentration (mg N/L), NO_2−_ = nitrites (mg N/L), NO_3−_ = nitrates (mg N/L), Kjeldahl N = Kjeldahl nitrogen (mg N/L), Org N = organic nitrogen (mg N/L), Total N = total nitrogen (mg N/L), PO_4_^3−^ = orthophosphates (mg P/L), Total P = total phosphorous (mg P/L). Only significant results are shown.

Mayfly Taxa/Abiotic Parameter	BOD	COD	NH_4_^+^	NO^2−^	NO^3−^	Kjeldahl N	Org N	Total N	PO_4_^3−^	Total P
*Baetis fuscatus*				R = 0.24,				R = 0.41,	R = 0.39,	R = 0.46,
				*p* = 0.05				*p* = 0.0003	*p* = 0.001	*p* = 0.0001
*Baetis buceratus*	R = 0.29,	R = 0.27,	R = 0.40,		R = 0.51,			R = 0.38,	R = 0.45,	
	*p* = 0.02	*p* = 0.03	*p* = 0.001		*p* = 0.000			*p* = 0.001	*p* = 0.000	
*Baetis rhodani*				R = −0.46,	R = −0.28,			R = −0.48,	R = −0.30,	R = −0.32,
				*p* = 0.0001	*p* = 0.02			*p* = 0.0002	*p* = 0.01	*p* = 0.01
*Baetis vernus*				R = −0.26,		R = −0.24,	R = −0.33,			
				*p* = 0.03		*p* = 0.04	*p* = 0.01			
*Baetis liebenauae*								R = 0.25,		
								*p* = 0.03		
*Baetis lutheri*		R = −0.25,								
		*p* = 0.04								
*Cloeon dipterum*								R = 0.25,		
								*p* = 0.03		
*Procloeon bifidum*								R = 0.23,		
								*p* = 0.05		
*Procloeon pennulatum*						R = −0.30,	R = −0.27,			
						*p* = 0.01	*p* = 0.02			
*Electrogena ujhelyii*				R = −0.50,		R = −0.37,		R = −0.52,	R = −0.54,	R = −0.61,
				*p* = 0.000		*p* = 0.001		*p* = 0.000	*p* = 0.000	*p* = 0.000
*Heptagenia longicauda*				R = 0.30,				R = 0.28,	R = 0.28,	R = 0.24,
				*p* = 0.01				*p* = 0.02	*p* = 0.02	*p* = 0.04
*Habrophlebia lauta*				R = −0.32,		R = −0.29,	R = −0.30,	R = −0.25,		R = −0.36,
				*p* = 0.01		*p* = 0.01	*p* = 0.01	*p* = 0.03		*p* = 0.002
*Ephemera lineata*				R = 0.25,						
				*p* = 0.03						
*Potamanthus luteus*					R = 0.25,			R = 0.40,	R = 0.32,	R = 0.26,
					*p* = 0.04			*p* = 0.001	*p* = 0.01	*p* = 0.03
*Serratella ignita*				R = 0.38,				R = 0.40,	R = 0.29,	
				*p* = 0.001				*p* = 0.001	*p* = 0.02	
*Caenis luctuosa*		R = 0.25,			R = 0.25,			R = 0.33,	R = 0.34,	R = 0.33,
		*p* = 0.04			*p* = 0.04			*p* = 0.01	*p* = 0.004	*p* = 0.01
*Caenis* cf. *pseudorivulorum*				R = 0.24,	R = 0.38,			R = 0.45,	R = 0.31,	
				*p* = 0.04	*p* = 0.001			*p* = 0.0001	*p* = 0.01	

**Table 5:** Relationship of mayfly species abundance and distance from source, land-use category, and morphological modification in a Pannonian lowland river. Positive correlations are shaded. Only significant results are shown.

Mayfly Taxa/Environmental Variables	Distance from Source	Land-Use Category Share (%)				Morphological Modification
		Near Natural	Intensive Agriculture	Extensive Agriculture	Urban	
*Baetis fuscatus*	R = 0.65,		R = −0.37,	R = 0.59,	R = 0.44,	
	*p* = 0.000		*p* = 0.001	*p* = 0.000	*p* = 0.0001	
*Baetis buceratus*	R = 0.27,	R = −0.31, *p* = 0.001			R = 0.30,	
	*p* = 0.01				*p* = 0.01	
*Baetis rhodani*	R = −0.41,	R = 0.27,			R = −0.36,	R = 0.27,
	*p* = 0.0004	*p* = 0.02			*p* = 0.002	*p* = 0.02
*Baetis vernus*					R = −0.23,	
					*p* = 0.05	
*Centroptilum luteolum*				R = −0.27,		
				*p* = 0.02		
*Cloeon dipterum*						R = 0.29,
						*p* = 0.01
*Procloeon pennulatum*		R = −0.25,				
		*p* = 0.04				
*Electrogena ujhelyii*	R = −0.66,		R = 0.52,	R = −0.51,	R = −0.70,	
	*p* = 0.000		*p* = 0.000	*p* = 0.000	*p* = 0.000	
*Heptagenia longicauda*	R = 0.36,					
	*p* = 0.002					
*Habrophlebia lauta*	R = −0.34,				R = −0.32,	
	*p* = 0.003				*p* = 0.01	
*Ephemera danica*					R = −0.24,	
					*p* = 0.04	
*Ephemera lineata*	R = 0.26,					
	*p* = 0.03					
*Potamanthus luteus*	R = 0.37,					
	*p* = 0.001					
*Serratella ignita*	R = 0.54,		R = −0.24,	R = 0.52,		
	*p* = 0.000		*p* = 0.04	*p* = 0.000		
*Caenis luctuosa*	R = 0.44,			R = 0.24,	R = 0.37,	
	*p* = 0.0001			*p* = 0.05	*p* = 0.001	
*Caenis* cf. *pseudorivulorum*	R = 0.50,	R = −0.39,			R = 0.37,	
	*p* = 0.000	*p* = 0.001			*p* = 0.002	

## Data Availability

The data presented in this study are available on request from the authors.

## Supplementary Materials

The following supporting information can be downloaded at: https://www.mdpi.com/article/10.3390/insects13050436/s1, Table S1: Dominant microhabitats (covering > 5% of sampling area) sampled at each study site. Legend: MACRO = Macrolithal (coarse blocks), MESO = Mesolithal (hand-sized cobbles), MICRO = Microlithal (coarse gravel), AKAL = Akal (fine to medium-sized gravel), PSA = Psammal (sand), ARG = Argyllal (clay), PHY = phytal (macrophytes; submerged and emergent), XYL = Xylal (woody debris), CPOM = deposits of coarse particulate organic matter, TECHNO = Technolithal (artificial blocks). The sampled phytal (macrophytes) is as follows: Study site 2-emergent reeds and emergent broad-leaved angiosperms. Study site 7-algae and fine-leaved submerged angiosperms. Study site 11-submerged fine-leaved angiosperms. Study site 17-emergent reeds and amphibious angiosperms. * Technolithal covered with moss.
