# Supplementary material for: The Impact of Hydromorphological Alterations on Mayfly Assemblages of a Mid-Sized Lowland River in South-Eastern Europe
Source: Insects. 2022 May 6;13(5):436. doi: 10.3390/insects13050436 (PMC9146438; doi:10.3390/insects13050436)
Supplement: Supplementary file 1 [file insects-13-00436-s001.zip › insects-1704881-SI.pdf]

Supplementary Table S1. Dominant microhabitats (covering > 5% of sampling area) sampled at each study site. Legend: MACRO = Macrolithal (coarse blocks), MESO = Mesolithal (hand-sized cobbles), MICRO = Microlithal (coarse gravel), AKAL = Akal (fine to medium-sized gravel), PSA = Psammal (sand), ARG = Argyllal (clay), PHY = phytal (macrophytes; submerged and emergent), XYL = Xylal (woody debris), CPOM = deposits of coarse particulate organic matter, TECHNO = Technolithal (artificial blocks). The sampled phytal (macrophytes) is as follows: Study site 2 - emergent reeds and emergent broad-leaved angiosperms. Study site 7 - algae and fine-leaved submerged angiosperms. Study site 11 - submerged fine-leaved angiosperms. Study site 17 - emergent reeds and amphibious angiosperms. *Technolithal covered with moss.

**Study site / microhabitat (%)**	**1**	**2**	**3**	**4**	**5**	**6**	**7**	**8**	**9**	**10**	**11**	**12**	**13**	**14**	**15**	**16**	**17**	**18**	**19**	**20**
MACRO	10%					10%														
MESO	35%		20%			75%										50%		30%		
MICRO	15%		30%	5%		10%	55%				20%	15%	10%			10%		30%	50%	70%
AKAL	5%	20%	10%			5%	10%	80%	80%	95%		55%	50%	55%	85%	15%		20%		5%
PSA	25%	40%	5%	50%	45%			5%	10%			20%	20%	40%	10%	5%				15%
ARG	10%		30%		40%															
PHY		40%					5%				25%						5%			
XYL				45%	15%			15%	10%	5%		10%	20%	5%	5%	20%		20%		10%
CPOM			5%																	
TECHNO							30%				55%*						95%		50%	
